# Improved performance of district health systems through implementing health center clinical and administrative standards in the Amhara region of Ethiopia

**DOI:** 10.1186/s12913-019-3939-y

**Published:** 2019-02-19

**Authors:** Mesele Damte Argaw, Binyam Fekadu Desta, Temesgen Ayehu Bele, Abebe Dagnew Ayne

**Affiliations:** 1 USAID Transform: Primary Health Care, JSI Research & Training Institute, Inc. in Ethiopia, P. O. Box 1392, 1110 Addis Ababa, Ethiopia; 2 Federal Ministry of Health, Health Extension and Primary Health Services Directorate, Addis Ababa, Ethiopia; 3 Amhara Regional State Health Bureau, Disease Prevention and Health Promotion Core Process, Bahir Dar, Ethiopia

**Keywords:** Standards, Health center reform, Performance improvement, Primary health care, EHCRIGs

## Abstract

**Background:**

Standards represent benchmarks against which improvements can be measured. In 2016, the Federal Ministry of Health developed and endorsed a set of standards entitled the Ethiopian Health Center Reform Implementation Guidelines (EHCRIGs). This study aims to assess the effects of planned interventions on performance and quality of services in primary health care units (PHCUs).

**Methods:**

A quasi-experimental pre-post study design was used to compare the performance of PHCUs against the ECHRIG standards before and after its implementation in 76 woredas of the Amhara region from July – December 2017. Pre and post-intervention validation measurements of performance improvement in 76 woredas were conducted. The data were entered and analyzed using Statistical Package for Social Sciences.

**Result:**

For this study, ten sessions of two-day trainings were conducted for 1306 staff. Additionally, on-site mentoring and coaching was conducted for a period of three months. The average EHCRIG standards met before and after intervention totaled 59 and 66%, respectively. The scores showed a positive correlation coefficient (*r* = 0.74) with statistical significant paired sample t-test with *t* = − 7.15, df = 75, *P* < 0.000. In addition, the mean scores among performance tiers were 74, 66 and 65% for high, medium and low performing woredas respectively. The One-way Analysis of Variance (ANOVA) showed a borderline significant difference between groups with (F = 2.4, *P* < 0.09).

**Conclusions:**

The implementation of the standards has garnered significant improvements in performance at the primary health care level. Therefore, continuing the initiated strengthening of health system performance against the standards and evaluation of the quality of primary health services is recommended.

**Electronic supplementary material:**

The online version of this article (10.1186/s12913-019-3939-y) contains supplementary material, which is available to authorized users.

## Background

The World Health Organization (WHO 2007) defines health systems as: “The sum total of all the organizations, institutions and resources whose primary purpose is to improve health.” [1] These entities need to provide services that are responsive and financially fair while treating people decently. Globally, limited available resources and increasing healthcare expenditure with high rates of service demand have amplified the need for institutionalizing innovative performance standards, performance measurements and performance improvements. Reporting progresses have also become key elements of ensuring accountability through exercising planning, contracting, directing, implementing, monitoring and evaluating activities [2, 3]. Like many developing countries, Ethiopia strives to strengthen its health systems through implementing several initiatives and interventions of WHO’s recommended six building blocks, namely: Service Delivery; Health Workforce; Information; Medical Products, Vaccines, and Technologies; Financing; and Leadership and Governance [1].

In the last two decades, the Government of Ethiopia has made significant gains in improving the health status of its citizens. The life expectancy of its citizens improved from 45 years in 1990 to 64 years in 2014; maternal mortality ratio has declined from 1400 deaths per 100,000 live births to 351 in 2016 and under-five mortality rate has declined by 67%, to 68 deaths per 1000 births [4]. These results were achieved as a result of strong commitment from the Ethiopian government as well as technical and other resource support from development partners. Hence, the Ministry is in a favorable position to create demand and to expand access to effective interventions to improve maternal, newborn and child health outcomes.

Despite these gains, the government recognizes the need to maintain progress and tackle the high maternal and neonatal mortality rates. In addition, it works to narrow the variability among regions, districts (woredas) and primary health care facilities. As providing quality health services in an equitable manner is a major goal, developing clinical and administrative standards are a key element of health reform interventions.

Professional Standards Review Council (1974) as cited in Donabedian (1981) defines standards as: “Professionally developed expressions of the range of acceptable variation from a norm or criterion.” In addition, in medical care, norms are numerical or statistical measures of observed performance, and criteria are predetermined elements against which aspects of the quality of medical service may be compared [5]. A practice standard is a statement that defines the performance expectations, structures, or processes that must be in place for an organization to provide safe and high-quality care, treatment, and services [5]. In 2016, the Federal Ministry of Health (FMOH) of Ethiopia developed and endorsed minimum clinical and administrative performance standards called the Ethiopian Health Center Reform Implementation Guidelines (EHCRIGs). These standards are expected to be adhered to by all primary health care units [6].

According to Armstrong (2009), performance management is defined as: “A systematic process for improving organizational performance by developing the performance of individuals and teams. It is a means of getting better results by understanding and managing performance within an agreed framework of planned goals, standards and competency requirements.” [7] In addition, Weiss and Hartle (1997) as cited in Armstrong (2009) define, performance management as: “A process for establishing a shared understanding about what is to be achieved and how it is to be achieved and an approach to managing people that increases the probability of achieving success.” [7] Furthermore, performance improvement and quality improvement are addressed using data collected from probing: “How can one ensure whether the changes bring the desired improvement?”. Reporting progress deals with “Communicating results to broader audiences”. Therefore, performance management involves a systematic and structured problem-solving approach and demands a conducive environment that encompasses transparency, visible leadership, customer focus, strategic alignment and culture of quality [3].

Through the Health Sector Transformation Plan (2015–2020), the Ethiopian government’s Ministry of Health aims to significantly improve access and quality of universal health service coverage through implementing several sector-wide reforms, and innovative performance management and improvement interventions [4]. Hence, a range of health reforms were undertaken to cultivate a culture of working against standards and enhance team work among the health workforce in Ethiopia. The Federal Ministry of Health and Regional Health Bureaus believe that compliance with standards like EHCRIGs will contribute to improving the effectiveness and empowerment of health workforces and streamlining decision making [6]. Therefore, the initiative helps to ensure that health service managers carry out their roles and responsibilities through improving their effectiveness and assists performance review teams to guide primary health care facility staff to become more efficient in their roles.

### Institutionalizing clinical and administrative guidelines

USAID Transform: Primary Health Care is a five-year project (January 2017 – December 2021), funded by United States Agency for International Development (USAID) [8]. The project is a successor of an eight year project, Integrated Family Health Program (IFHP) (2008–2016) which had a very large geographic reach and improved the lives of millions of women, men and young people throughout Ethiopia through implementing interventions in 301 woredas located in Amhara; Oromia; Southern Nations, Nationalities and Peoples (SNNP) and Tigray regions as well as a few woredas in Benshangul Gumuz and Somali Regions [9]. Based on selected key maternal and child health indicators, 40 (13%), 95 (32%) and 165 (55%) of targeted districts were high, medium and low performing, respectively [8]. USAID Transform: Primary Health Care project provides technical and financial resource support towards the implementation of the Health Sector Transformation Strategic Plan (HSTPII) in four regional states of Ethiopia, namely; Amhara, Oromia, Southern Nations Nationalities and Peoples (SNNP), and Tigray. Moreover, within these regions, there are over 300 districts (woredas) receiving technical, financial and other resource support from the project [4, 8].

### The ‘theory of change’ in USAID transform: Primary health care project interventions

As a pathway to the agreed upon global targets for 2030 and beyond, the USAID Transform: Primary Health Care project, was developed to contribute towards the successful achievement of the national goal of preventing child and maternal deaths (PCMD). In addition, its long-term outcomes (LTO) are increased utilization of quality family planning and reproductive health, maternal newborn health, child health and development, adolescent and youth health and development and nutrition (RMNCAH- N) services under a well-functioning primary health care system [4].

**Fig. 1:**
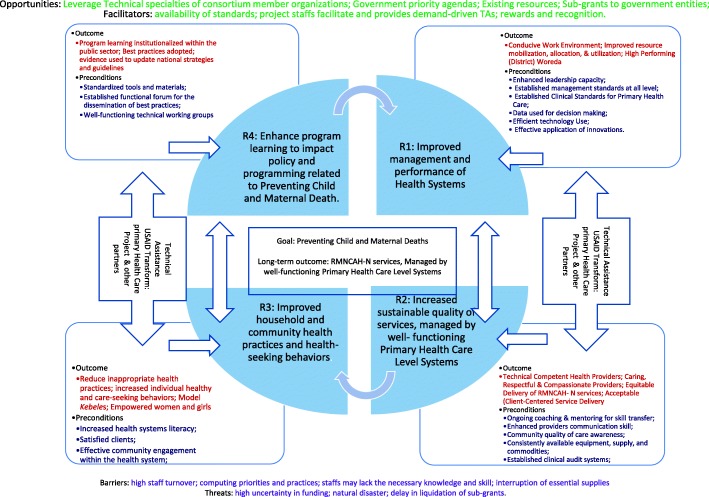
A diagrammatic presentation of the causal pathways [4]. The figure illustrates the theory of change with, Long term outcome, Long term outcomes, preconditions, and assumptions

In order to achieve the above goal, the project hypothesized four major preconditions and assumptions:

1. Successful achievement in creating a conducive work environment, improving resource mobilization, allocation, and utilization; and working towards high performing woredas will improve management and performance of health systems.
2. Enhanced the technical competence of health providers and enhancement of their skills will lead to increased sustainable quality of services, which is managed by well-functioning primary health care level systems.
3. Reduced inappropriate health practices will improve household and community health practices and health-seeking behaviors.
4. Program learning institutionalized within the public health sector will enhance program learning to impact policy and programs related to preventing child and maternal deaths.
  The theory of change is presented below using illustrations of the causal pathways (Fig. 1).

To strengthen health systems, from July to December 2017, USAID Transform: Primary Health Care project implemented performance management and improvement interventions in 76 (42%) districts in the Amhara region. A range of measures were taken to ensure that the district’s health system managers execute identified minimum standards effectively. These include; provision of training on the standards, self-assessment and validation of measurements, performance improvement, and reporting progress, coaching, observing, providing feedback and developing agreed action plans. The aim of this study was to document the effects of implementing clinical and administrative standards set in the Ethiopian Health Center Reform Guidelines on the performance of district (woreda) health systems. Therefore, this article highlights performance improvements using clinical and administrative standards as a result of USAID Transform: Primary Health Care project’s pre and post-intervention measurements of the Ethiopian Health Center Reform Implementation in the Amhara region of Ethiopia.

## Methods

### Study site

**Fig. 2:**
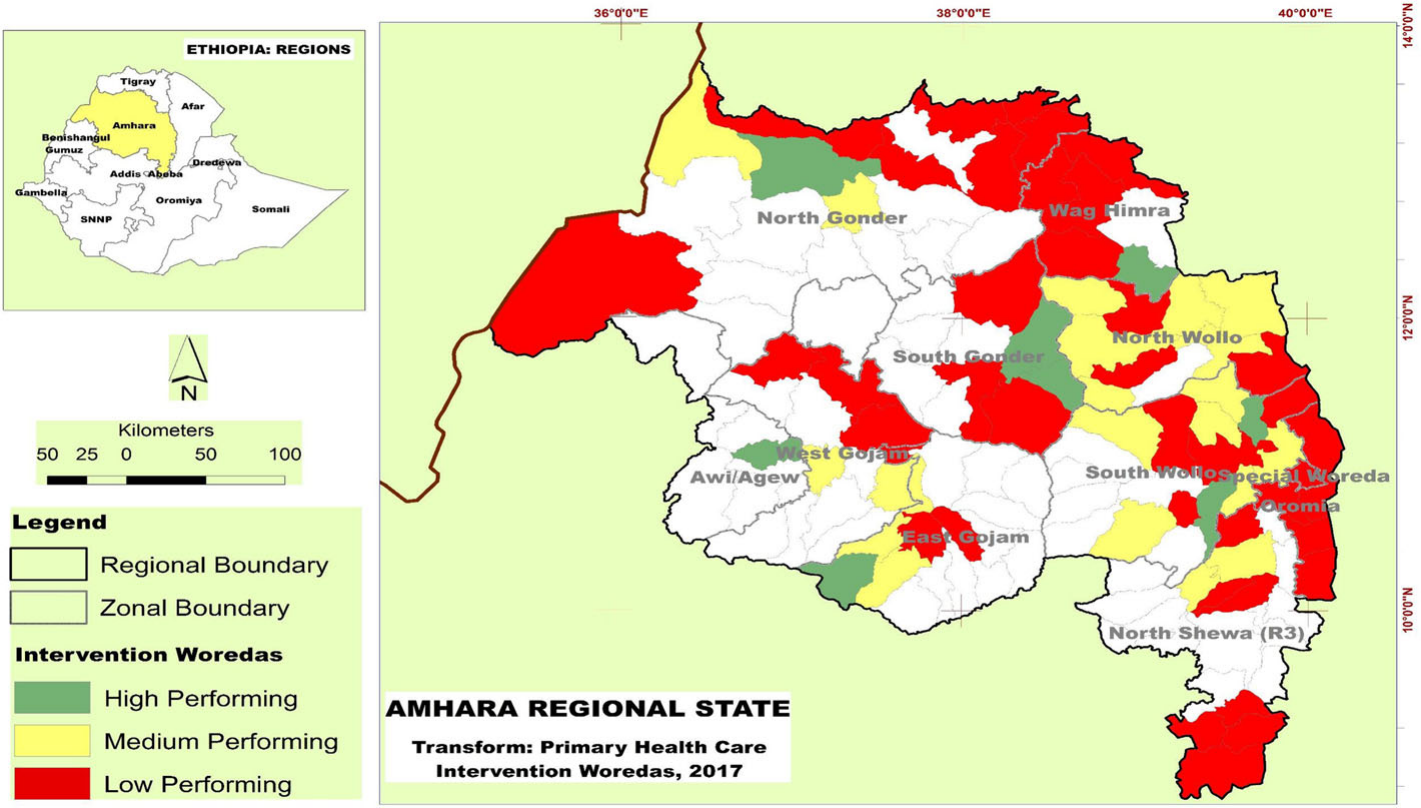
Map of the Study Area. Depicted map of the study area. The map clearly presented location of Amhara Region in Ethiopia and label performance tier of selected woredas

Ethiopia is located in the horn of Africa. The land area is estimated to be about 1.1million square kilometers [10]. Amhara region has a land mass of 154,709 km^2^ and lies within the geographic coordinate 11.3494° N, 37.9785° E. It has ten administrative zones and 181 districts (woredas). This study was conducted in purposively selected 76 (42%) districts, of which 10 were high performing, 23 were medium performing and 43 were low performing woredas (Fig. 2). USAID Transform: Primary Health Care project provides demand-driven technical, financial and resource support to strengthen the six building blocks of the health system. In addition, there are integrated service deliveries implemented in the areas of health systems strengthening, family planning and reproductive health, maternal newborn health, obstetric fistula, child health and development, expanded immunization program, nutrition, adolescent and youth health and development, malaria, gender, community-based health insurance and social and behavioral change communication.

### Health tier system calibers

**Fig. 3:**
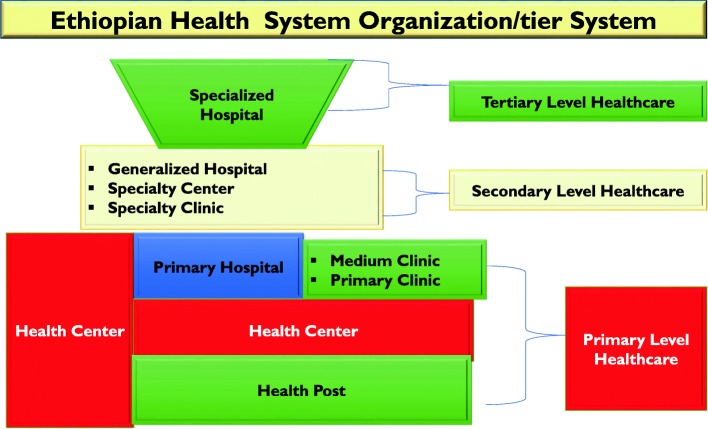
Ethiopian Health Tier System [12]. Depicted the Ethiopian Health Tier System, Where Health Centres are located at the primary level health care category

The Ethiopian health tier system has three levels of healthcare delivery systems, namely: primary, secondary and tertiary level of healthcare organizations. As depicted in the Fig. 3 below, level one is district (woreda) health systems comprised of primary hospitals which cover 60,000–100,000 people, health centers serving 15,000–25,000 people and their satellite health posts covering 3000–5000 people connected to each other through a referral system. According to the Ethiopian Service Standards [11] health center shall mean a health facility at the primary level of the healthcare system which provides promotive, preventive, curative and rehabilitative outpatient care including basic laboratory and pharmacy services with the capacity of 10 beds for emergency and delivery services. The primary hospitals, health centers and health posts form primary health care units (PHCUs). At level two are general hospitals covering a population of 1–1.5 million people, and at level three are specialized hospitals covering a population of 3.5–5 million people [12].

### Performance tier

Based on routine health management information system reports, twelve maternal and child health-related service indicators were selected and collated. A group of experts which consisted of the Federal Ministry of Health, USAID and IFHP rated achievements in accordance with the indicators with scores out of 100%. The key indicators included; early institutional neonatal mortality rate, proportion of surviving infants vaccinated for Penta-3, proportion of people tested positive for malaria: all ages, proportion of people tested positive for malaria: < 5 yrs., proportion of children with moderate malnutrition, proportion of children with severe malnutrition, institutional maternal death rate, proportion of pregnant women that received antenatal care: at least four visits, proportion of births attended by a skilled health worker, early postnatal care coverage, proportion of pregnant women tested for HIV, and contraceptive acceptance rate. Following scoring based on these criteria, the top one-third are categorized as high performer woredas, the middle one-third as medium performing woredas and the lower one-third are categorized as low performing woredas [8].

### Study design

A quasi-experimental pre-post design study was conducted among 76 districts of the Amhara region with technical and resources support being provided by USAID Transform: Primary Health Care project from July to December 2017 [13].

### Performance management and improvement intervention

The Amhara Regional Health Bureau provided training of trainers and validation of team members trainings from 20th to 22nd July 2017 in Bahir Dar City, Ethiopia. The trainings were then cascaded to all ten zones of the region in August 2017 (Additional file 1, Fig. 4). In each district (woreda), twenty-five staff members of health offices and health centers were trained on the standards of EHCRIGs, systematic problem-solving tools i.e. fishbone analysis and the ‘five why’ techniques, provision of support supervision and observation and validation techniques. In addition, each district selected one lead person and four members in primary health care facilities to report progress and organize learning collaboratives (i.e. quarterly organized field visits and performance review meetings). Furthermore, USAID Transform: Primary Health Care project staff and experts of zone health departments received the necessary orientation in implementing the standards in selected districts. Validation team members were deployed from September 1–25, 2017, to take preintervention measurements. Two sessions of onsite coaching were conducted on a quarterly basis. During facility visits, the teams inquired about the achievement of each health facility and ask a series of questions to get a holistic picture of the processes adapted to achieve the set standards. Through the discussions with primary health care facility teams, the coaches identified gaps and assisted the teams to strategically analyze the problems. In addition, the coaches provided feedback on performances and gave technical updates and information on guidelines deemed helpful to improve skill gaps. Following that, both the coaches and primary health care performance review (management) teams developed an agreed action plan on identified, prioritized solutions. The coaches then submitted a copy of agreed measurements reports, identified gaps and developed action plans to district health offices and the zone health department. Six months after the intervention which took place from 1 to 20^,^ April 2018, the validation team collected post intervention measurements.

**Fig. 4:**
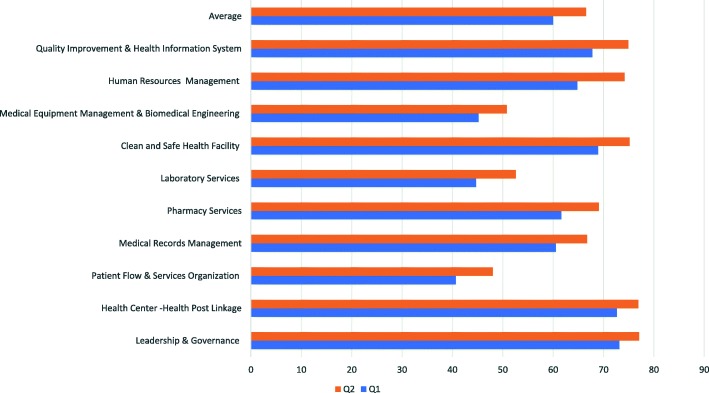
Pre and Post-intervention average score by ten chapters, December 2017. The figure showed that the pre and post intervention average score of 76 districts on each chapter

### Sampling

All 76 USAID Transform: Primary Health Care project intervention woredas were purposively selected.

### Data collection methods and data quality

Nationally developed and validated instruments and the Ethiopian Health Center Reform Implementation Guideline’s tools were adapted for this study. The pre-post interventions data collection tools have ten chapters and 81 standards with composite validation indicators. The tools were used to assess the availability of essential resources and services. The checklist used at primary healthcare facilities consists of ten chapters: (1) Leadership and Governance; (2) Health Center and Health Post Linkage; (3) Patient Flow and Service Organization; (4) Medical Records Management; (5) Pharmacy Services; (6) Laboratory Services; (7) Clean, and Safe Health Facility; (8) Medical Equipment Management and Biomedical Engineering; (9) Human Resources Management; and (10) Quality Improvement and Health Information System. Detailed description is presented in Table 1 below.

**Table 1:** Description of standards and indicators, EHCRIGs 2016 [6]

Chapter	Description of the indicators & composite criteria considered
Leadership and Governance	This chapter deals with evidence of establishment & functionality of how boards will be scored: i.e. minutes, official assignment; guidelines; term of references; regular board meetings; strategic and annual plans; established functional management committees; review performance of health center directors; post services costs; agreements signed to provide credit services; financial reports using balance sheets; approved purchase plans; financial guidelines; & audit reports. (12 standards and 35 validation criteria).
Health Center & Health Post Linkage	This chapter deals with scores to measure health center and health post linkages. The standards include, availability of health center and health post linkage guidelines, assigned focal person, agreed action plans with budgets, establishment of women development groups and 1 to 5 network of health development army, facilitation of supportive supervision on a weekly base, monthly capacity enhancement & performance review meetings; and availability of tracer drugs in the health posts. (8 standards with 300 validation criteria)
Patient Flow & Service Organization	The patient flow and service organization chapter has six standards and 20 validation criteria. The detail of the standards includes: developed protocol and procedures on patient flow, triage, referral and preparations to manage emergency services, training of staff to implement protocols, equipping of liaison officer, telephone facilities, registers, books, referral directory, appointment register, clear labels and/or signage of facilities and services, establishment of maternal homes (waiting area) with full sets of facilities: shower; toilets; kitchen etc. (6 standards with 20 validation criteria)
Medical Records Management	The medical records management chapter deals with the establishment of a well-organized archive room, institutionalization of unique patient identifiers, establishment of digital or manual index and availability of essential resources like cards forms, printed sheets, confidentiality of patient information through protocol and process, compliance officers, creating staff awareness on developed processes and arrange experience sharing event. (4 standards with 9 validation criteria)
Pharmacy Services	The pharmacy service chapter is designed to meet the primary needs of all customers. The detail criteria include establishment of a drug and therapeutic committee with Term of References, developed annual plan, testimony with minutes and reports, pharmacy department led by a pharmacist and store managed by a diploma level pharmacy technician, development of facility level drug list using VEN categorization, forecasting, purchasing & disposal guidelines, dispensing of drugs after recording detailed information of patients and drugs, establishment of a drug information center, protocol for management of side effects and other problems, use of logistics management tools, inventory of drugs and disposal guidelines, availability of tracer drugs in health posts, preparation of standard rooms and an audit report. (13 standards with 21 validation criteria)
Laboratory Services	This chapter deals with provision of laboratory services. Some of the standards refer to public information availibility about the services, time, and costs, counseling services with clients to understand the meaning of the investigations & results, preparation of adequate rooms, assignment of human resources and supplies as per the requirements and standards, institutionalization of laboratory operating management systems, availability of standard operating procedures (SOP) on sample collection, transport; storing & disposals, safety procedures, diagnostic algorisms, laboratory information systems, maintenance procedures of lab equipment, safety procedures in place, e.g. fire extinguisher and trained staff on safety, data safety & confidentiality, internal quality control and participation in External Quality Assurance.(9 standards with 23 validation criteria)
Clean, and Safe Health Facility	These standards measure the health facility’s engagement and dedication to providing services in a clean, safe and healthy environment, assignment of a focal person to facilitate technical support to all staff, allocation of budget, institutionalization of a functional infection prevention committee i.e. minutes, action plan and feedback, and availability of personal protective equipment, soaps; detergents; mops and hand tools to prepare land for gardening, support to health posts in implementing standard infection principles in their facilities, all staff training on infection prevention, health education to clients and patients on infection preventions, following of procedures of waste management, and adherence to medical instrument decontamination and sterilization procedures. (10 standards with 13 validation criteria)
Medical Equipment Management & Biomedical Engineering	The biomedical engineering chapter deals with the availability of functional medical equipment in the health facilities., Some of the standards are: inventory of medical equipment, functionality of new equipment, orientation to staff on use and care new equipment, prepare maintain request forms, maintenance of equipment, functionality and safety of medical equipment, availability of water and electricity supply i 24 h a day/seven days week. (8 standards with 40 validation criteria)
Human Resources Management	The human resource chapter deals with human resource management and development. The standards are; availability of human resource personnel, archiving of personal files with JD, employment letter and other testimonies, human resource development plan, establishment of motivation and reward systems, appraisal of the performance of health workforce every six months, use uniform and Identification cards for all staff. (6 standards with 8 validation criteria)
Quality Improvement and Health Information System	This chapter deals with quality improvement and complies with the routine Health Information System requirements. The standards are: establishment of quality improvement team, (minutes, Term of References), sharing of work plan to departments and staff aggregated by weeks months and quarters, implementation of quality improvement tools in selected health services i.e. problem-solving tools, adherence to routine health information system requirements timely, complete and consistent report submission and use of data for decision making, engagement of the community towards quality improvement and completion of client satisfaction and other surveys. (5 standards with 10 validation criteria)

Data were collected using structured questionnaires. The questionnaires were prepared in Amharic, the local and official national language. In each facility, two to five health professionals were trained on the standards, measurements, performance improvements and reporting progress tools. In addition, the validation teams recruited from zone health departments, project and partner staff provided trainings on the standards, criteria, observation techniques, ethical consideration as well as providing technical support to facility members.

### Data analysis

Standards measurement reports were submitted by trained health professionals working in health centers and districts (woreda) health offices. Data on average scores for district health offices were reviewed and data of district health offices measured on two occasions were included. For each district, data on performance across the ten chapters of EHCRIGs were entered in Microsoft Office Excel Spreadsheet (2010), then exported to Statistical Package for Social Science Research (SPSS IBM Version 20) for analysis [14]. The data collected from district health offices were cleaned and checked for consistencies then described using frequencies, proportion, and percentages and were presented using charts and tables. Based on the proportion of standards met, scores out of a hundred were used to categorize districts into three performance levels i.e. ideal best performing primary health care facility which met greater or equal to 80% of standards; mid performing primary health care facility for scores ranging from 60 to 79.9% and low performing primary health care facility for scores below 60% of the standards [6]. In addition, to determine the linear relationship between pre and post intervention scores, the Pearson Product-Moment Correlation technique was employed; then a paired t-test was employed to estimate the mean score difference in district health office performances between the first and the second measurement scores. To check for statistical difference in performance tier of targeted districts, the test used in this study was one-way analysis of variance (ANOVA). A statistically significant relationship was claimed at *P* < 0.05.

### Ethical clearance

Ethical clearance was obtained from the Amhara National Regional State Health Bureau Research and Ethics Committee. Permission to use the data was sought and obtained from the USAID Transform: Primary Health Care project. In this study, no facility identifier information was collected. The summary of this study was synthesized and submitted to the regional health bureau on two occasions. Moreover, the district health system managers were encouraged to use the evidence for performance management and improvement.

## Results

### Distribution of districts with primary health care units

This study analyzed the pre-post intervention validation measurement in EHCRIGs standards in 76 woredas of the Amhara region. As is indicated in the table below, within ten administrative zones of Amhara region, the number of districts that received direct support from USAID Transform: Primary Health Care project range from the least 2 to the highest 14. On average, the number of functional primary health care units were 5.8 ± (SD) 2.3 (Table 2).

**Table 2:** Distribution of districts (woredas) and health centers in the study area

Zone	Number of Woredas	Proportion	Number of Health Centers	Proportion
Awi Zone	2	3%	10	2%
Wag-Himira	5	7%	23	5%
Oromia	6	8%	26	6%
East Gojjam	6	8%	32	7%
North Shoa	10	13%	38	9%
South Gondar	6	8%	52	12%
West Gojjam	8	11%	57	13%
Gondar	10	13%	59	13%
North Wollo	9	12%	62	14%
South Wollo	14	18%	84	19%
Total	76	100%	443	100%

NB: * Health Centers Mean ± Standard Deviations (SD) = 5.8 ± 2.3

### Socio-demographic background of trained health professionals

As depicted in the table above, the implementation of the EHCRIGs was started with the provision of training for 1306 health professionals. Among these, 207 (15.9%) were female. With regards to the professional mix of the trainees, the largest 395 (30.2%) were health information technicians; followed by diploma nurses 344 (26.4%) and 5 (0.4%) possessed a BSc in Midwifery (Table 3).

**Table 3:** Socio-demographic characteristics of trained professionals, July –December 2017

Characteristics	Frequency	Percent (%)
Sex		
Male	1099	84.1%
Female	207	15.9%
Total	1306	100%
Professions		
BSc in Midwifery Nursing	5	0.4%
Pharmacist (B Pharm)	6	0.4%
Diploma Pharmacy Technicians	26	2.0%
BSc in Environmental Health Officers	31	2.4%
Master’s in public health	38	2.9%
Diploma Medical Laboratory Technicians	46	3.5%
Diploma in Midwifery Nursing	50	3.8%
BSc Nurses	101	7.7%
Public Health Officers	264	20.2%
Diploma Nurses	344	26.4%
Health Information Technicians	395	30.2%
Total	1306	100%

### Changes in EHCRIG standard performances

The validation pre-post intervention measurement showed the proportion of met standards increased by 6% in all chapters. The majority of district health systems scored very close to the threshold (80%) in four chapters of the categorization of high performing primary health care facilities. During the intervention period, there were improvements in the average of standards met such as: (73 to 77%) in leadership and governance, (73 to 77%) in health center-health post linkage; (69 to 75%) in clean, and safe health facility and (68 to 75%) in quality improvement and health information systems chapters. Conversely, the performance scores in three chapters were close to 50%; namely: (41 to48%) in patient flow and service organization; (45 to 53%) in laboratory services; and (45 to 51%) in medical equipment management and biomedical engineering chapters (Fig. 4).

### Changes in district health system performance

The district health systems improved their performance as a result of USAID Transform: Primary Health Care project’s technical and resource support interventions. The majority of the districts scored medium at the second validation measurement with a mean score of 66% and a range between 36 to 90% compared to the first quarter measurement scores of 59% mean and range of 35 to 89%. The difference between the first and second measurements scores was 7% and was statistically significant (t = − 7.15, df = 75, *P* = 0.000). However, five districts showed a decline in performance scores met against standards. Table 4 Summarizes the district health system performance scores across the first and second validation scores.

**Table 4:** Comparison between first and second validation measurement scores using paired t-test

Variables	Mean Score (%) ± SD	Minimum Score (%)	Maximum Score (%)
First Score (baseline)	59 ± 11.86	35	89
Second Score	66 ± 12.12	36	90
Difference between first and second validation measurement score	7 (t = −7.15, df = 75, P = 0.000), r = 0.74		

Note: Total number of district health offices is 76; SD: Standard Deviations

As presented, the result of descriptive statistics below (Table 5), the mean scores with Standard Deviations (SD) were 74.3 ± 10.7; 66.0 ± 12.0; and 66.6 ± 12.1% of EHCRIGs which were documented for high; medium and low performing districts (woredas), respectively. Resultts revealed that there were high performing districts with the minimum score of 60.0% and a maximum score of 90% and low performing districts with a minimum score of 36% and followed by 89% for maximum scores.

**Table 5:** Comparison of descriptive statistics of EHCRIGs scores by district performance tiers

Performance tier & number of Woredas	Mean	SD	Std. Error	95% Confidence Interval for Mean		Minimum	Maximum
				Lower Bound	Upper Bound		
High (10)	74.3	10.7	3.4	66.6	82.0	60.0	90.0
Medium (23)	66.0	12.0	2.5	60.8	71.2	44.0	85.0
Low (43)	65.2	12.1	1.8	61.5	68.9	36.0	89.0
Total (76)	66.6	12.1	1.4	63.9	69.4	36.0	90.0

As the results show in the table below (Table 6), one-way ANOVA was employed to test mean differences of EHCRIGs scores and district (woreda) performance tiers. The results revealed that EHCRIGs mean scores have a borderline statistical significance relationship with district performance tiers (F(2, 73) = 2.4, *P* = 0.09).

**Table 6:** Analysis of Variance (One-way ANOVA) between EHCRIGs scores and district (woreda) performance tier

Source of Variances		Sum of Squares	df	Mean Square	F	Sig.
Woreda (district) performance tier	Between Groups	680.9	2	340.4	2.4	0.09
	Within Groups	10,350.0	73	141.8	–	–
	Total	11,030.9	75	–	–	–

### EHCRIG performance categorization zones by chapters using scorecard

Based on the national criteria to categorize district health systems using EHCRIGs scores, districts scored greater or equal to 80, 60 to 79.9% and less than 60% were classified as high performing, medium performing and low performing district health systems, respectively. For this study, developed scorecards were comprised of ten chapters and 81 standards with 209 composite indicators. The green, yellow and red coded dashboard showed relative performance scores of each zone’s administrations. Presented in the table below are the relative differences in pre and post intervention achievements against each chapter of the scorecards for each zone. The majority of the green coded performances were achieved by Wag Himira, Awi, South Wollo and East Gojjam zones. Red coded performances were observed in three zones, namely: North Wollo, Oromia, and North Shoa. Despite relatively increased achievement in all chapters, in three chapters, i.e. patient flow and organization; laboratory services and biomedical engineering standards scored red-coded level of achievements (Table 7), and the overall performance scores ranged from 76% in Wag-Himira to 63% in the Oromia zone.

**Table 7:**
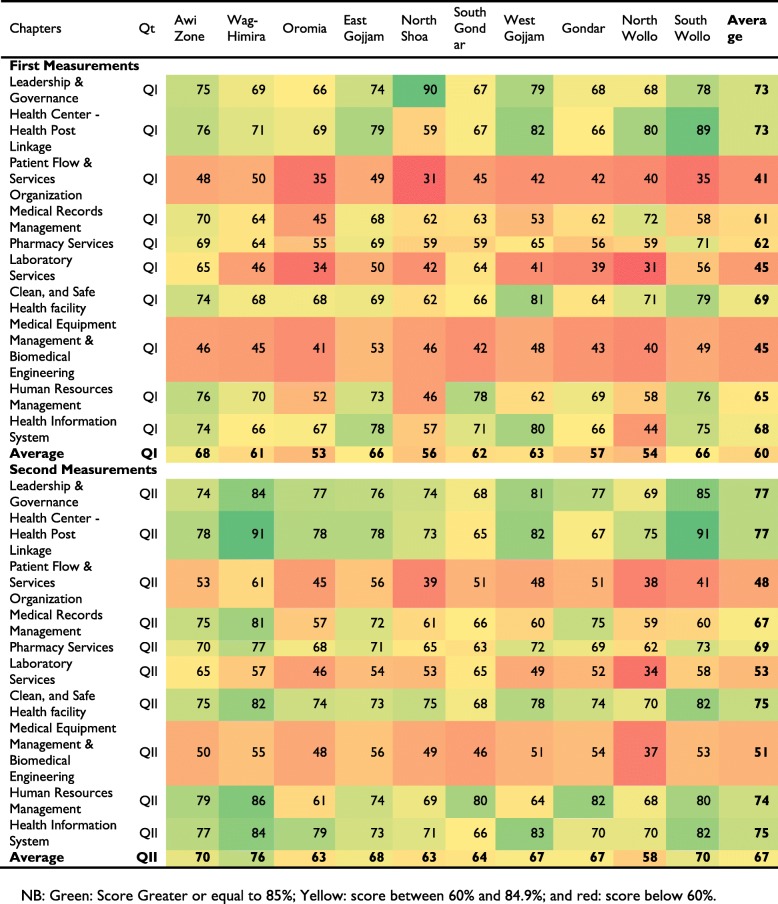
Scorecard of performance scores by chapters and zones

## Discussion

This pre-post intervention survey highlighted the importance of performance improvement through implementing clinical and administrative standards to strengthen district health systems. The data were collected in 76 woredas within the Amhara region of Ethiopia. All sample primary health care facilities and district health systems submitted pre and post intervention measurements. The response rate was 100%. The high response rate could be due to an intensive involvement of the public health sector, development partners and other relevant stakeholders. In addition, the direct technical and resource support from USAID Transform: Primary Health Care project, zone health departments, and other development partners consisting of orientations on standards, performance measurements, performance improvement through problem-solving, and action plan development and organizing reporting on progress is likely to positively influence participation and response rates.

Furthermore, the leadership of Amhara regional state’s health bureau took the initiative to exercise the standards and use the performance measurement scores for recognition and rewards using semi-annual, result presentation workshops. The main purpose of the implementation of the health center reform standards was to improve performance and quality of care at the district health system level using ten chapters and 81 standards of the EHCRIGs [6]. Generally, the results of this study revealed a positive correlation, coefficient with statistically significant differences in mean performance scores between pre and post intervention stages. These significant performance improvements were achieved through the execution of several interventions within six months.

The project provided tailored technical, financial and other resource support which enabled the district health system managers to create a conducive work environment for health workforces. In addition, through the involvement of stakeholders, the project enhanced capacity in use of systematic problem-solving tools, developing agreed action plans, and ensured appropriate measures and actions were taken. This approach improved the culture of working in teams, created shared visions and understanding of the mission of organizations, helped in mobilizing and aligning the necessary resources as well as implementing and following up with monitoring the performance of district health systems.

With the support of the project, the technical competency of health care providers was enhanced through coaching and arranging several experiences sharing and learning collaborative workshops. Trained coaches and validation team members facilitated meetings and strived to develop the knowledge and skills of performance management teams within the district health systems. This finding was in line with Bailey et al. (2016) systematic review report which confers supportive supervision causes increased job satisfaction and increases health workers’ motivation [15]. Furthermore, the project strived to institutionalize social accountability and implemented health system strengthening initiatives so that the community would receive safe and quality health services at the primary health care level. In addition, the technical support of the project ensured the presence of guideline tools to reduce inappropriate health practices.

Regarding program learning initiatives, USAID Transform: Primary Health Care project enhanced the capacity of district health system managers to facilitate review meetings and document case studies through the use of zone health department and project staff. Prior to support from the project, performance review meetings were not effective and efficient. Following project’s efforts to change this, the number of review meeting participants ranged from 100 to 150 with attendants comprised of kebele managers, health extension workers as well as health center and district health office staff. Each health facility attending the meetings presents plans versus achievement using over 122 indicators. Moreover, the discussion sessions address a range of issues including staff training and development as well as transfer. Subsequent meetings are arranged at the end of each review meeting prior to forming action plans. Through USAID Transform: Primary Health Care project’s guidance, the Ethiopian Primary Health Care Alliance for Quality (EPAQ) was adhered to ensuring that the number of participants was limited to 25 to 30 [16]. The participants include managers who are directors of the health center, board chair and health center-health post linkage focal person who are responsible for scanning, focusing, planning, organizing, aligning and mobilizing, implementing, monitoring and evaluating health system strengthening activities. Agenda of meetings consist of 5 chapters of woreda (district) Management Standards; 10 chapters of the EHCRIGs; 29 to 37 selected Key Performance Indicators (KPIs) and five change package achievements. In addition, experience sharing, and validation of reports are arranged among staff of lead and member facilities. Finally, action plans were developed on identified priority activities with a responsible person, time and resources allotted. This new approach helps health managers to improve their performance against the standards within the district health system. Such purposeful review meetings and access to facilitative supervisors documented elsewhere were shown to improve achievements of health facilities in low and middle-income countries [17–24].

The majority of district health system performance management scores were significantly improved in four chapters, i.e. Leadership and Governance; Health Center-Health Post linkage; Clean, and Safe Health Facility; and Quality Improvement and Health Information Systems. This result could be due to the technical and financial support provided to successfully implement change packages [16, 25]. Despite all the technical and on-site support, the scores for three chapters remained low i.e. Patient Flow and Service Organization, Laboratory Services and Biomedical Engineering standards. The low scores could be due to these chapters and standards demanding a high level of technical skills. In addition, lack of properly trained human resources in the primary health care facilities could negatively affect the performance of district health systems. Furthermore, to successfully meet these standards, primary health facilities should be fulfilling the structural requirement [26] such as adequate spaces for counseling services, triage and storing which may require a significant budget. The gains in these chapters after six months of interventions ranged from 4 to 7%. This could be due to that some standards demand recruitment of staff and renovation of health facilities which can be costly as well as limited technical skills available at primary health care facilities.

### Limitations

This quasi experimental study had several limitations. The first limitation was that it only measured results after six months of interventions which means the long-term effects of the applied standards were not observed. In addition, the study did identify predictor variables. Finally, the opinions of staff on compliance with the standards were not included.

## Conclusions

The implementation of performance management improvement through implementing the Ethiopian Health Center Reform Implementation Guideline Standards and its tools significantly improved the performance of district health systems in the Amhara region of Ethiopia. Scaling up trainings, systematic problem-solving tools, coaching sessions, facilitating experience sharing and learning collaboratives as interventions in performance improvement of district health systems can be effective in enhancing the overall performance management. However, the findings revealed that three major chapters; (1) Patient Flow and Service Organization; (2) Laboratory Services; and (3) Medical Equipment Management and Biomedical Engineering Standards may require more specialized, technical, financial and other resource support since the validation scores were lowest for these indicators. In addition, continuing with health system strengthening interventions through implementing service standards and improvements in quality of primary health care services is recommended.

## Additional file.

Additional file 1: Performance assessment checklists with eighty one standards its validation composite criteria. (XLSX 32 kb)

## Abbreviations

ANOVA: Analysis of Variance
EHCRIGs: Ethiopian Health Center Reform Implementation Guidelines
EPAQ: Ethiopian Primary Health Care Alliance for Quality
FMOH: Federal Ministry of Health
HSTP: Health Sector Transformation Plan
IFHP: Integrated Family Health Program
KPIs: Key Performance Indicators
USAID: United States Agency for International Development
RHB: Regional Health Bureau
WHO: World Health Organization

## References

[1] WHO (2007). Everybody’s business--strengthening health systems to improve health outcomes: WHO's framework for action.

[2] Hall W, Smith N, Mitton C, Urquhart B, Bryan S (2018). Assessing and improving performance: a longitudinal evaluation of priority setting and resource allocation in a Canadian health region. Int J Health Policy Manag.

[3] Armbruster S, McCarty A, Moran J. Guide to the stages of performance management: A guide to help health departments make progress toward a culture of performance management. Washington: Public Health Foundation; 2018.

[4] FMOH (2015). Health sector transformation plan.

[5] Donabedian A (1981). Criteria, norms and standards of quality: what do they mean?. Am J Public Health.

[6] FMOH (2016). Ethiopian health centers reform implementation guidelines.

[7] Armstrong M (2009). Armstrong’s handbook of human resource management practice.

[8] USAID Transform: Primary Health Care Project. Theory of change in practice 2017. Addis Ababa: USAID Transform: Primary Health Care; 2017.

[9] IFHP (2016). End of program report 2008–2016.

[10] Central Statistical Agency [Ethiopia] and ICF International. Ethiopia Demographic and Health Survey 2011. Addis Ababa, Ethiopia and Calverton, Maryland, USA: Central Statistical Agency and ICF International; 2012.

[11] FMHACA (2012). Health center requirement. Addis Ababa. Food. Medicine and health care administration.

[12] FMOH (2010). Health sector development program IV (2010/2011–2014/2015).

[13] Thiese MS (2014). Observational and interventional study design types; an overview. Biochemia medica: Biochemia medica.

[14] SPSS Inc. IBM SPSS statistics base 20. Chicago, IL: SPSS Inc.; 2011.

[15] Bailey C, Blake C, Schriver M, Cubaka VK, Thomas T, Martin Hilber A (2016). A systematic review of supportive supervision as a strategy to improve primary healthcare services in sub-Saharan Africa. Int J Gynecol Obstet.

[16] FMOH (2017). Ethiopian primary health care Alliance of quality (EPAQ).

[17] Marquez L, Kean L (2002). Making supervision supportive and sustainable: new approaches to old problems. Popul Rep.

[18] Adair CE, Simpson E, Casebeer AL, Birdsell JM, Hayden KA, Lewis S (2006). Performance measurement in healthcare: part I–concepts and trends from a state of the science review. Healthcare Policy.

[19] Suter E, Oelke ND, Adair CE, Armitage GD. Ten key principles for successful health systems integration. Healthcare quarterly (Toronto, Ont.). 2009;13(Spec No):16. 10.12927/hcq.2009.21092 PMC3004930 20057244

[20] Lutwama GW, Roos JH, Dolamo BL (2013). Assessing the implementation of performance management of health care workers in Uganda. BMC Health Serv Res.

[21] Andrews R, Boyne GA, Moon MJ, Walker RM (2010). Assessing organizational performance: exploring differences between internal and external measures. Int Public Manag J.

[22] Trap B, Ladwar DO, Oteba MO, Embrey M, Khalid M, Wagner AK. Article 1: supervision, performance assessment, and recognition strategy (SPARS)-a multipronged intervention strategy for strengthening medicines management in Uganda: method presentation and facility performance at baseline. J Pharmaceut Policy Pract 2016;9(1):21. 10.1186/s40545-016-0070-x PMC4888637 27252869

[23] Tadesse DM, Demissie HF, Segni MT (2015). Improvement in Adherence to Ethiopian Hospitals Reform Implementation Guideline nursing standards in Asella Referral Teaching Hospital: a pre-post study. Int Med Publ J.

[24] Madede T, Sidat M, McAuliffe E, Patricio SR, Uduma O, Galligan M, Bradley S, Cambe I (2017). The impact of a supportive supervision intervention on health workers in Niassa, Mozambique: a cluster-controlled trial. Hum Resour Health.

[25] Gulelat, B. Assessment on the implementation of Hospital Reform Guideline with Reference to Pharmacy service in Addis Ababa: Addis Ababa University; 2014. (master’s thesis).

[26] Kringos DS, Boerma WG, Hutchinson A, Van der Zee J, Groenewegen PP (2010). The breadth of primary care: a systematic literature review of its core dimensions. BMC Health Serv Res.

